# From Dust to Blood: Studies Predict Lead Intake in Children

**Published:** 2009-03

**Authors:** Rebecca Kessler

Lead concentrations in U.S. children’s blood have decreased markedly in recent decades, thanks largely to lower industrial emissions, voluntary elimination of lead solder in food cans, and legislation barring lead from gasoline and new paint. The main source of lead exposure for today’s children is deteriorating lead-based paint, which contributes to lead-laden dust in older homes. Two studies, the first of their kind to use nationally representative data from U.S. homes, predict how varying degrees of lead contamination of floor and windowsill dust may affect the blood lead levels of resident children **[*EHP* 117:461–467; Gaitens et al.; *EHP* 117:468–474; Dixon et al.]**.

Despite reductions in child blood lead levels, the U.S. Centers for Disease Control and Prevention (CDC) estimates on the basis of 1999–2002 data that some 310,000 children still have levels above the agency’s threshold of concern, 10 μg/dL. Such children are at increased risk for cognitive impairment and behavioral problems. Mounting evidence [e.g., *EHP* 116:243–248 (2008)] has linked even lower blood lead levels with adverse effects.

The current studies examined lead- and housing-related data for a nationally representative group of 2,155 children aged 1–5 years, drawn from the National Health and Nutrition Examination Survey (NHANES) from 1999 through 2004. In addition to blood lead data, dust samples had been collected from floors and windowsills in the children’s homes and analyzed for lead content. The study by Gaitens et al. showed that dust lead levels in the great majority of homes met or exceeded federal standards: just 0.16% of homes failed the standard for floors of 40 μg/ft^2^, and 4.0% failed the standard for windowsills of 250 μg/ft^2^. Income, race/ethnicity, floor condition, windowsill dust lead content, year of home construction, recent renovation, smoking, and survey year all were significant predictors of floor dust lead loading, which was more predictive than windowsill dust lead of elevated blood lead in residents.

Dixon et al. examined blood lead levels for the same 2,155 children and used a linear regression model to predict children’s blood lead given a range of floor dust lead concentrations from very low (0.25 μg/ft^2^) up to the federal standard of 40 μg/ft^2^. Based on logistic regression models, the authors estimated that among children living in pre-1978 homes with floor dust lead levels of 12 μg/ft^2^, 4.6% would have a blood lead level of at least 10 μg/dL, whereas 27% would have a level of at least 5 μg/dL. Because the blood lead and dust lead levels observed in the NHANES data set were relatively low, the researchers verified the models’ predictive capacity by analyzing data from three high-risk populations with higher levels of both blood lead and floor dust lead than those observed in NHANES.

The studies indicate that most U.S. homes already meet federal standards for floor and windowsill dust lead levels, but also suggest that further tightening of the standards would afford greater protection for today’s children. However, although data for both studies came from a nationally representative sample of children, the homes may not necessarily represent the U.S. housing stock. The authors cite the need for an integrated health and housing survey that is representative of both the population and the housing stock, similar to surveys recently conducted in Europe.

